# Expiration Date of Extemporaneous Pyrimethamine Formulations for Treating Congenital Toxoplasmosis: Physicochemical and Microbiological Evaluation

**DOI:** 10.1021/acsomega.5c12714

**Published:** 2026-06-02

**Authors:** Renato Medeiros de Paula, Vitoria Beatriz Silva Santana, Marcelo Vítor de Paiva Amorim, Douglas Dourado, Éverton do Nascimento Alencar, Débora Araújo Othon de Aquino, Lourena Mafra Veríssimo

**Affiliations:** † Department of Pharmacy, 28123 Federal University of Rio Grande do Norte (UFRN), Natal, Rio Grande 59078-970, Brazil; ‡ University for International Integration of the Afro-Brazilian Lusophony (UNILAB), José Franco de Oliveira Street, Redenção, Ceará 62.790-970, Brazil; § College of Pharmaceutical Sciences, Food and Nutrition, 54534 Federal University of Mato Grosso do Sul (UFMS), Campo Grande, Mato Grosso do Sul 79070-900, Brazil; ∥ Center for Food and Drug Research (NUPLAM), UFRN, Natal, Rio Grande 59078-900, Brazil

## Abstract

This study evaluated the stability of extemporaneous pyrimethamine oral suspensions prepared from commercial tablets using 85% sucrose or 70% sorbitol as vehicles. A validated HPLC method was employed to quantify pyrimethamine and assess its chemical stability. Physicochemical parameters including macroscopic appearance, pH, particle size, and drug content were monitored for 90 days at 5 and 25 °C. Both formulations maintained acceptable pH values and pyrimethamine content within pharmacopeial limits. Particle size increased over time, likely due to aggregation, while appearance remained unchanged for up to 45 days. Microbiological assessments showed that microorganisms remained within acceptable limits, with no objectionable microorganisms detected. Overall, both suspensions demonstrated chemical and microbiological stability for up to 45 days (Suspension S) and 60 days (Suspension X) under both storage conditions. These results support the preparation of cost-effective pyrimethamine formulations for treating congenital toxoplasmosis in pediatric patients.

## Graphical abstract

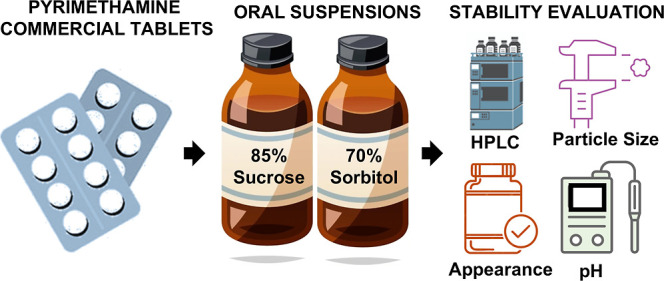

## Introduction

Toxoplasmosis is a globally prevalent zoonotic disease caused by *Toxoplasma gondii*, a unicellular eukaryotic parasite. This disease presents a complex epidemiology with a wide range of clinical manifestation^1,2^ . *T. gondii* exhibits the highest prevalence rates in tropical regions of Africa, Southeast Asia, the Middle East, Central and Eastern Europe, and Latin America. The infection rates vary widely across different regions: Asia (13.3%–85.3%), Europe (40%–76%), Africa (21.74%–74.8%), and North America (7.3%–26.5%)^2–5^ .

Acute infection by *T. gondii* during pregnancy can lead to a range of maternal and fetal complications. The main clinical concern, however, is the vertical transmission of the parasite to the fetus, with transmission rates ranging from 20% to 50% in the absence of treatment. Congenital toxoplasmosis (CT) may result from a primary infection acquired during pregnancy, reactivation of latent infection in immunocompromised women, or reinfection with more virulent strains of the parasite. CT can result in long-term behavioral and neurocognitive impairments, as well as visual deficits, significantly compromising the quality of life, particularly among untreated children^4,6^ .

The standard treatment for CT in pediatric patients under one year of age involves the concurrent administration of sulfadiazine (100 mg/kg/day), pyrimethamine (2 mg/kg/day), and folinic acid (15 mg).^4^ Among these drugs, pyrimethamine, which acts by inhibiting the folate biosynthesis pathway, is considered the principal agent responsible for the therapeutic efficacy of the regimen.^7^ However, in the pediatric context, the administration of the drug requires suitable pharmaceutical forms to ensure proper adherence to the treatment, especially in young children^8^.

The recommended dose of pyrimethamine is 2 mg/kg/day, administered orally every 12 h.^6,7^ Currently, the available dosage form is in tablet form, which contains a fixed dose per unit. This format poses significant challenges for the treatment of newborns, who cannot swallow tablets, and the required dose varies depending on the child’s weight.^4,8^

To address these challenges, extemporaneous suspensions prepared from tablets have become a common practice in hospitals, compounding pharmacies, and, concerningly, even at home. Although liquid dosage forms are more suitable for pediatric patients, these extemporaneous formulations are often not prepared under proper water quality, hygiene, and dose accuracy standards. In the case of CT, the stability of these suspensions is unknown, which can compromise the efficacy of treatment, especially considering the need for prolonged treatments. While compounding pharmacies may provide stable formulations, their use is limited due to high costs.^9^

From a pharmaceutical standpoint, the preparation of extemporaneous suspensions presents several challenges, particularly in maintaining the physicochemical stability of the active pharmaceutical ingredient.^10^ In the case of pyrimethamine, its low aqueous solubility despite high membrane permeability places it in Biopharmaceutical Classification System (BCS) Class II, requiring careful attention to formulation strategies to enhance its dispersion and bioavailability in aqueous media.^11^

Additionally, the selection of appropriate vehicles, the achievement of uniform dispersion, and the assurance of microbiological stability are critical for ensuring both the safety and therapeutic efficacy of the formulation in pediatric patients.^12,13^ The unique physiological characteristics of children, including immature gastrointestinal and metabolic systems, further impact drug pharmacokinetics, underscoring the need for precise dosing and consistent administration to avoid subtherapeutic effects or toxicity.^14^

Considering the challenges in pediatric treatment, this study aims to prepare extemporaneous oral suspensions by converting commercially available pyrimethamine tablets into a liquid dosage form using two different vehicles (syrup or sorbitol) and storage conditions (room temperature and refrigeration). Furthermore, it will evaluate the physicochemical and microbiological stability of these formulations over time. The results will provide essential data to guide healthcare professionals, helping them to prepare stable and effective formulations to ensure the safe and effective treatment of CT in pediatric patients.

## Results and Discussion

### Formulation Production

Two extemporaneous suspensions were produced from pyrimethamine tablets. Suspension S used 70% sorbitol as the vehicle, while Suspension X used 85% of sucrose (simple syrup) as vehicle. The vehicle and/or the characteristics of the tablets may influence the visual appearance of the extemporaneous product. Suspension S appeared as white fluid, while Suspension X had a light beige color. Both suspensions displayed colors compatible with the starting white tablets, the colorless sorbitol solution, and the light beige sucrose solution. Furthermore, the formulations were characterized, as displayed in the following sections. Initially, the HPLC method was validated for proper pyrimethamine quantification.^15^

### Method Validation

Analytical method validation is a globally recognized process for ensuring that pharmaceutical products demonstrate minimal, well-defined, and reproducible deviations. It enables the safe and consistent manufacturing of products in terms of content and other physicochemical properties over time and across different locations. Consequently, method validation is a mandatory requirement from regulatory agencies in drug research and production.^16,17^

The analytical method proposed in this study for the HPLC quantification of pyrimethamine was adapted from the USP method, which was originally developed for suspensions based on simple syrup.^18^ In addition, further methods have been proposed for pyrimethamine quantification from (i) tablets^15^ (ii) powders^19^ (iii) mixtures of other drugs^20^ (iv) and blood.^21^ However, the USP method was not fully applicable to the formulations in this study due to the use of different excipients. Consequently, the method described here was adapted (mobile phase composition, injection volume, detection wavelength, chromatographic column and flow rate) and based on the USP method, but it was validated before use.

The specificity of the method was evaluated to ensure the purity of the pyrimethamine peak, considering potential degradation products and excipients from the tablets and vehicles. The method demonstrated a peak purity of 1.0, a resolution above 2.0, and no interference from other peaks at the 13 min retention time of pyrimethamine, as shown in Figure 1 below.

**Figure 1:**
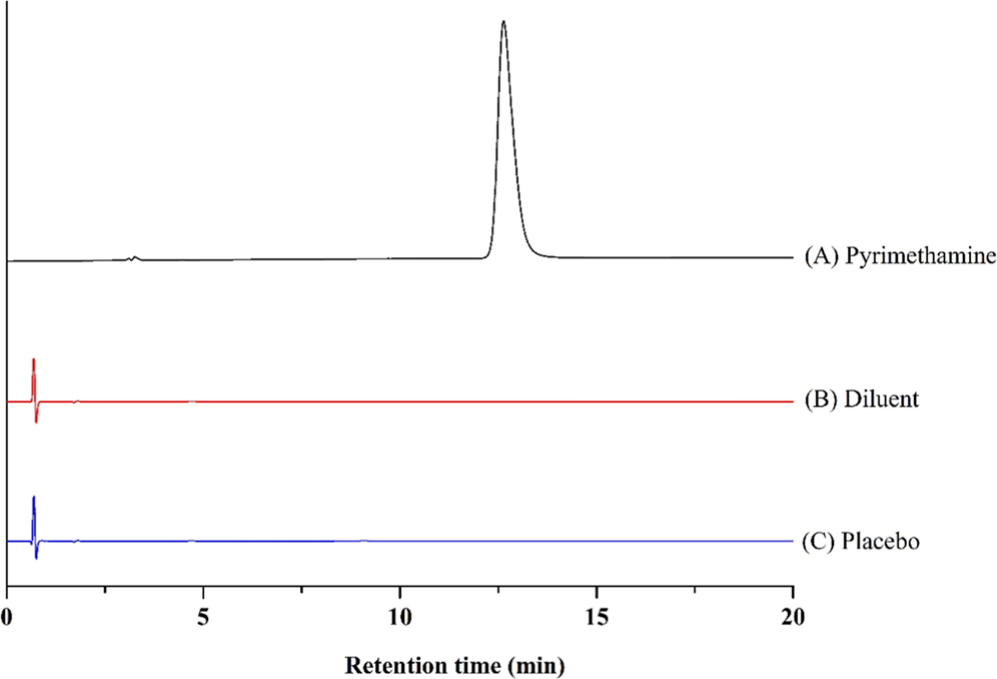
Representative chromatograms demonstrating the specificity of the HPLC method. Peaks correspond to (A) standard pyrimethamine solution; (B) diluent blank; and (C) placebo formulation.

These results confirm the method’s specificity for the samples used in this study. Forced degradation studies of pyrimethamine suspensions under various conditions (acidic and basic hydrolysis) showed no interference with peak purity (Figure 2). Specifically, pyrimethamine Suspension X (simple syrup) exhibited approximately 10% degradation under acid hydrolysis conditions, while the Suspension S (70% sorbitol) showed less than 10% degradation. Both suspensions maintained peak purity above 0.99. Therefore, it was demonstrated that the method is specific and suitable for assessing the stability of pyrimethamine in different vehicles over time.^22^

**Figure 2:**
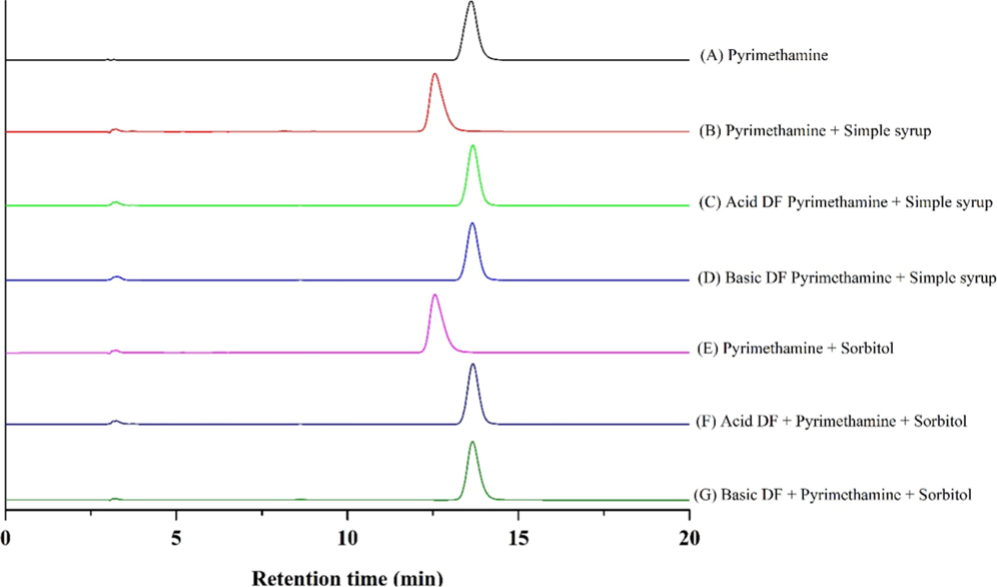
Representative chromatograms demonstrating the selectivity of the HPLC method under stress conditions of acid and base hydrolysis. Peaks correspond to (A) standard pyrimethamine solution; (B) pyrimethamine in simple syrup formulation; (C) pyrimethamine in simple syrup after acid degradation; (D) pyrimethamine in simple syrup after base degradation; (E) pyrimethamine in sorbitol formulation; (F) pyrimethamine in sorbitol after acid degradation; and (G) pyrimethamine in sorbitol after base degradation.

The linearity of an analytical method refers to the proportional relationship between output signals and concentration levels. In this study, linearity was evaluated through a calibration curve developed in triplicate, represented by the equation *y* = 69072*x*–166,748, which ranged from 80% to 120% of the target concentration (12.8 μg/mL to 19.2 μg/mL). The method demonstrated strong linearity, as evidenced by a correlation coefficient (*R*) of 0.9990 within the tested concentration range.^23^

Precision was assessed through intermediate precision and repeatability. The method exhibited acceptable repeatability for both samples, with Suspensions X and S showing RSD of 1.77% and 1.79%, respectively. All RSD values met the criteria established by the USP 41-NF 36 (no more than 2.0%). The intermediate precision study also yielded RSD values below 2.0% for both samples, indicating that the developed method can be reliably executed across different days and by different analysts while maintaining reproducibility.^24^

Accuracy was evaluated based on recovery percentage, which reflects the closeness of the measured outputs to the true values. As summarized in Table 1, both Suspensions exhibited recovery percentages ranging from 99% to 102% for all tested concentrations. The RSD values remained within the 2% acceptance criteria, with the highest RSD for Suspension X of 1.28% and 1.05% for Suspension S.

**Table 1:** Accuracy of the HPLC Method Expressed as Recovery Percentage, Demonstrating the Closeness of the Measured Values to the True Concentrations of Pyrimethamine

	concentration (%)	target concentration (mg/mL)	recovered concentration (mg/mL)	recovery (%)	RSD (%)	confidence Interval (±)
suspension X	80	0.0128	0.0127	99.23	1.28	0.000010
	100	0.0160	0.0160	100.20	0.53	0.000015
	120	0.0192	0.0191	99.33	0.40	0.000012
suspension S	80	0.0132	0.0134	101.65	0.67	0.000007
	100	0.0160	0.0160	100.00	1.05	0.000005
	120	0.0192	0.0193	100.63	0.15	0.000009

The robustness assay evaluates the method’s ability to maintain accuracy and reliability despite deliberate variations in analytical parameters.^17^ The evaluation is designed to test the method’s resilience to potential fluctuations during routine measurements, such as minor changes in flow rate, pH, and solvent proportions.

Tables 2 and 3 show the robust results for the quantification of Suspensions S and X, respectively. In addition to the suspensions, standard solutions were also injected for comparison. Key parameters, including tailing factors, resolution, theoretical plates, and capacity factors, were calculated for all injections. Regardless of the suspension vehicle used (sucrose or sorbitol) and variations in different parameters, all calculated values remained within the acceptance criteria. However, a decrease in the number of theoretical plates was observed in the suspensions compared to the standard solution, particularly when mobile phase composition varied.

**Table 2:** Robust Results of the Analytical Method for Quantifying Pyrimethamine Suspension S (70% Sorbitol)

suspension S						
	condition	solution	tail factor	theoretical plates	capacity factor	resolution
pH	2.5	standard	1.13	7180	3.64	2.67
		sample	1.14	5806	3.74	2.91
	original	standard	1.25	10,667	3.84	3.15
		sample	1.49	4160	3.75	3.05
	2.7	standard	1.12	4618	3.99	2.39
		sample	1.15	4216	3.97	3.82
flow rate	0.9	standard	1.32	11,772	4.31	3.37
		sample	1.51	40,020	4.20	2.55
	original	standard	1.25	10,667	3.84	3.15
		sample	1.49	41,600	3.75	3.05
	1.1	standard	1.23	10,768	3.35	3.39
		sample	1.54	41,230	4.20	5.85
mobile phase composition	58:42	standard	1.18	81,210	2.81	2.56
		sample	1.16	75,510	2.80	2.42
	original	standard	1.25	10,667	3.84	3.15
		sample	1.49	41,600	3.75	3.05
	62:38	standard	1.18	84,290	4.37	2.18
		sample	1.16	82,190	4.38	4.36
acceptance Criteria			<2.00	>4000	>2.00	≥2.00

**Table 3:** Robustness Results of the Analytical Method for Quantifying Pyrimethamine in Suspension X (Simple Syrup) 85%

suspension X						
	condition	solution	tail factor	theoretical plates	capacity factor	resolution
pH	2,5	standard	1.16	9899	2.96	2.95
		sample	1.19	9539	2.99	2.51
	original	standard	1.25	10,667	3.84	3.15
		sample	1.25	10,198	3.70	3.05
	2,7	standard	1.19	9820	3.26	2.90
		sample	1.17	9331	3.27	2.60
flow rate	0,9	standard	1.32	11,772	4.31	3.37
		sample	1.30	11,494	4.30	3.33
	original	standard	1.25	10,667	3.84	3.15
		sample	1.25	10,198	3.70	3.05
	1,1	standard	1.23	10,768	3.35	3.39
		sample	1.22	10,472	3.35	3.21
mobile phase composition	58:42	standard	1.53	4396	2.45	1.83
		sample	1.49	4267	2.45	1.67
	original	standard	1.25	10,667	3.84	3.15
		sample	1.25	10,198	3.70	3.05
	62:38	standard	1.20	5385	3.69	2.70
		sample	1.18	5537	3.68	2.90
acceptance criteria			<2.0	>4000	>2.0	≥2.0

Despite this, the required minimum number of theoretical plates was still met. Suspension X’s resolution for a mobile phase of 58:45 v/v fell below acceptance criteria, indicating a critical adjustment needed. Overall, variations in mobile phase pH and flow rate did not compromise the method’s ability to adequately separate the peaks. Additionally, the excipients in the suspensions, whether from the vehicle or the tablets, did not alter the results beyond the established acceptance criteria. Thus, the robustness assay confirmed that the method is capable of withstanding potential variations during sample quantification, except for mobile phase composition.

The evaluation of validation parameters confirmed that the method is suitable for quantifying pyrimethamine in suspensions with various vehicles. In line with ICH and USP guidelines, and meeting the acceptance criteria for specificity, linearity, precision, accuracy, and robustness, the HPLC method adapted in this study can accommodate minor analytical variations. It can be reliably used across different days and by different analysts while still accurately and reproducibly quantifying pyrimethamine in a range of extemporaneous suspensions.^15,25^

### Stability Study

#### Macroscopic Aspects of Extemporaneous Suspensions

The organoleptic characteristics of the suspensions remained unchanged for the initial 30 days (Table 4). However, after this period, Suspension X began to exhibit crystalline deposits in the storage vial at both storage temperatures. By day 60, the same suspension exhibited a difficult-to-redisperse precipitation. Similarly, Suspension S remained stable for the first 45 days but showed signs of instability by day 60, with macroscopic white aggregates forming and adhering to the vial cap, regardless of the storage temperature. This behavior corroborates the findings of Soares Rodrigues Costa et al.,^26^ who reported that sulfadiazine extemporaneous suspensions in the same vehicles exhibited similar instability characteristics beginning on day 14 of storage. This observation further supports the notion of instability in these formulations.

**Table 4:** Macroscopic Appearance of Pyrimethamine Extemporaneous Oral Suspensions^a^

day	suspension S (sorbitol 70%)	suspension X (sucrose 85%)
0	white, homogeneous fluid	light beige, homogeneous fluid
2	no change	no change
7	no change	no change
15	no change	no change
30	no change	no change
45	no change	crystalline deposits start forming
60	white aggregates at cap, less redispersible	dense precipitation, difficult to redisperse
90	visible white clumps, partial sedimentation	persistent sediment, irreversible precipitation

a All observations were consistent across storage at both 25 ± 2 °C and 5 ± 3 °C.

Although qualitative, the analyses of macroscopic and visual characteristics of extemporaneous suspensions offer valuable stability data that reflects the physicochemical properties of the raw materials used in the formulation. This is especially important because the technical characteristics of the original tablet change during the preparation of the suspension, leading to a loss of its inherent physicochemical properties. Hence, straightforward qualitative parameters like macroscopic and visual assessments help determine the shelf life of these formulations.^27^

Despite quantitative parameters, such as sedimentation volume/ratio, degree of flocculation, and rheological profile, are essential for determining overall suspension stability, extemporaneous suspensions, particularly in compounding practice, are only used for clinically safe periods. Hence, macroscopic, persistent, irreversible sediments (caking) are considered unsafe for homogeneous administration, unlike redispersable sediments, which can be considered nonlimiting if the drug content remains constant, primarily in the case of pharmaceutical adaptations.^19^ This analysis is further presented in this study, helping support overall formulation stability decisions.

### pH Analysis

pH is a crucial chemical parameter for liquid oral dosage forms, as it directly influences drug solubility, stability, and absorption. Variations in pH can significantly impact formulation stability by initiating or accelerating reaction rates that can lead to a loss of drug content. Consequently, this can result in reduced therapeutic efficacy of the product.^28^

The pH values of Suspensions X and S over 90 days are presented in Figure 3. Suspension X (Figure 3a), stored at 25 °C, exhibited an initial pH of 7.03 ± 0.03 on day 0. After 60 days, the pH decreased significantly to 6.5 ± 0.06. In contrast, the refrigerated sample, which had an initial pH of 6.98 ± 0.01, remained stable for a longer period, with a significant decrease (*p* < 0.05) to 6.14 ± 0.01 by day 90 of the analysis. Furthermore, Suspension S (Figure 3b) demonstrated a statistically significant variation (*p* < 0.05) during the period studied for both formulations stored at room temperature and under refrigeration.

**Figure 3:**
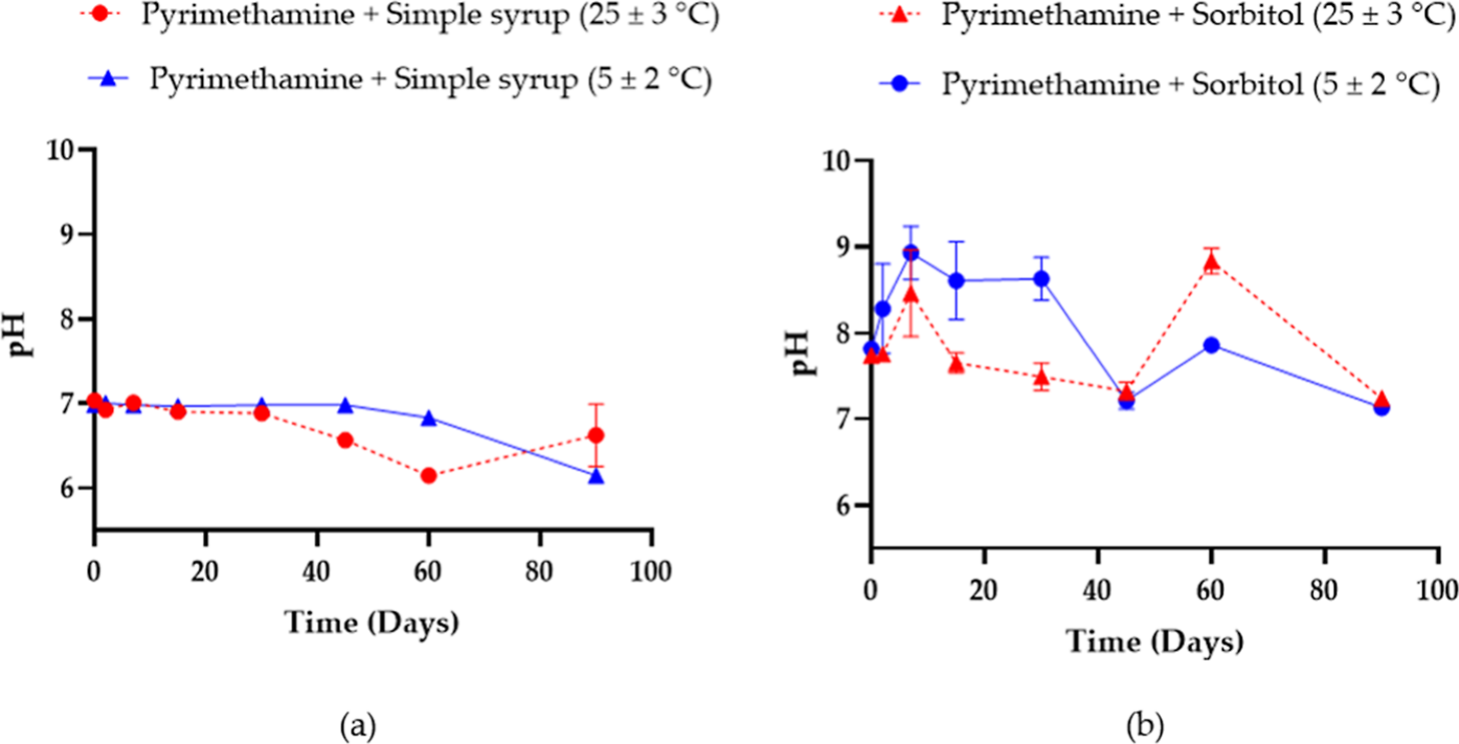
pH variation of extemporaneous pyrimethamine formulations over 90 days, stored under two temperature conditions: (a) simple syrup as the vehicle and (b) sorbitol solution as the vehicle. Samples were maintained at 25 ± 3 °C (dashed lines) and 5 ± 2 °C (solid lines). Data are presented as mean ± standard deviation (*n* = 3).

The vehicles used in the development of the aforementioned study are routinely employed in the pharmaceutical industry and hospital settings.^29^ It is understood that sucrose is a carbohydrate formed from the combination of glucose and fructose, while sorbitol is a naturally occurring sugar alcohol.^30^ Both substances are characterized by a neutral pH, as reported in the literature.^31^ However, initial findings of the solutions evaluated in a complementary manner without the influence of active ingredients and excipients on day zero revealed that sorbitol solution exhibited a pH of approximately 8.1 (±0.04) at both temperatures, whereas simple syrup showed an initial pH of approximately 6.0 (±0.01).

Hence, it is evident that the vehicles yielded values different from neutral pH. However, it is essential to consider factors such as the synthesis of the raw materials, the solvents used in the manufacturing process, the degree of purity, and the handling of both solutions, as these may lead to variations from a neutral pH.^28^ Additionally, the presence and quality of water used as a solvent should also be considered. Consequently, while the pH of the vehicles deviates from neutrality, it is crucial to consider the other components of the formulation, as described below.

Given that extemporaneous formulations contain excipients derived from tablets, pH control over time is a critical parameter. Unlike a simple suspension, extemporaneous suspensions incorporate various other excipients that may behave undesirably at different pH levels, thereby increasing the potential for drug instability. Furthermore, considering the inherent characteristics of vehicles, pH must be controlled to ensure that these compounds do not adversely affect the formulation. For instance, sucrose hydrolysis into glucose and fructose occurs at a faster rate under higher temperatures and lower pH values.^32^

The excipients present in the tablet include sodium docusate, which exhibits low acidity, and lactose monohydrate, magnesium stearate, and starch, all of which are neutral.^33^ In addition to the vehicle used in the extemporaneous formulation and the tablets’ excipients, it is important to consider the physicochemical properties of the active ingredient, even though it is not the predominant component of the formulation.^34^ Pyrimethamine, a crystalline salt with low water solubility, exhibits weak base properties due to its amine and aminomethyl groups, with an aminomethyl p*K*_a_ of 7.26. Consequently, when the pH exceeds this value, ionization occurs.^35,36^

Thus, the formulations primarily consist of neutral components. However, the initial pH values indicated a slight increase in sucrose and a slight decrease in sorbitol compared to the initial results of the solutions without the pulverized tablet components. This suggests that the powdered components influenced the pH of the suspension relative to its initial state without these additives.^29^

Regarding temperature, as previously mentioned, the pyrimethamine molecule behaves as a weak base, while all other components tend to be neutral. However, in this study, considering the properties of the vehicles, excipients, and active ingredients, neither refrigerated nor ambient storage temperatures demonstrated a direct impact on the pH of the formulation. It is important to note that pH variation with temperature is more pronounced in solutions containing stronger acids and bases.^28^

Overall, a slight pH decrease was observed for the suspensions throughout this study. However, this decrease is not inherently indicative of inadequacy for use, considering the extemporaneous nature of the formulation and the potential interactions between the tablet excipients and the suspension vehicle. The neutral to slightly acidic pH values obtained after 90 days remain appropriate for oral administration.^14^ Regarding the chemical and physicochemical impacts that pH could have on the suspension, a further investigation targeting drug content and particle size is described as follows.

### Particle Size

Optimal suspensions are characterized by uniform dispersion and the ability to form easily redispersable sediments when sedimentation occurs, allowing for resuspension through gentle shaking.^37^ As noted in previous sections, Suspensions X and S exhibited limitations in resuspension at days 45 and 60, respectively. Furthermore, no excipient optimization for sedimentation modulation was performed on these formulations, which reflects the real challenges faced in hospital and domestic settings. This limitation may affect therapeutic efficacy and directly impact the reproducibility of particle size measurements in suspensions.^38^

Particle size assessments were conducted on the suspensions over 90 days. Figure 4 summarizes the results from these assays. Suspension X (Figure 4a) exhibited an initial particle size of approximately 44 μm on day 0, consistent across samples stored at room temperature and those refrigerated. However, after 45 days of storage, the particle size changed significantly (*p* < 0.05) to 25.84 (±2.48) μm and 20.20 (±2.6) μm for samples stored at 25 ± 3 °C and 5 ± 3 °C, respectively. Notably, particle size did not vary significantly with temperature in the 85% sucrose solution. Despite this significant reduction at day 45, by day 90, the particle size of Suspension X at both temperatures had increased to approximately its initial size.

**Figure 4:**
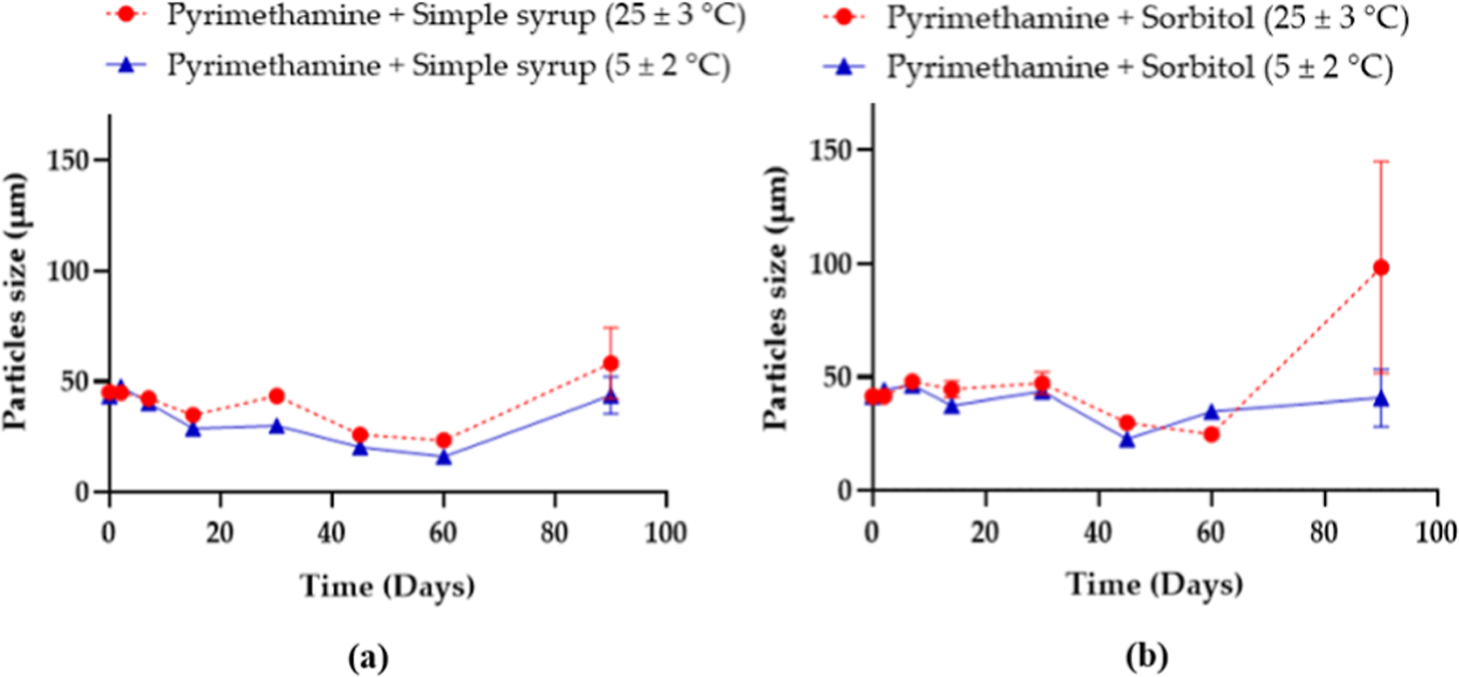
Particle size variation of extemporaneous pyrimethamine formulations over 90 days, stored under two temperature conditions: (a) simple syrup as the vehicle and (b) sorbitol solution as the vehicle. Samples were maintained at 25 ± 3 °C (dashed lines) and 5 ± 2 °C (solid lines). Data are presented as mean ± standard deviation (*n* = 3).

Similarly, suspension S (Figure 4b) showed an initial particle size of approximately 41 μm. After being stored at different temperatures, the particle size exhibited a significant difference (*p* < 0.05) at day 60 for the room temperature sample, measuring 24.65 (±0.9) μm, while the sample stored at 5 ± 3 °C measured 34.71 (±2.94) μm at the same time point. Overall, at day 30, both suspensions remained unchanged in terms of particle size across different storage temperatures. These findings corroborate the size behavior demonstrated in the work of Neis et al. 2024, wherein pyrimethamine suspensions resuspended by gentle manual shaking showed an initial particle size of 91.4 ± 4.00 μm, with no significant change after 30 days of storage (81.93 ± 9.88 μm, *p* > 0.05) stability.^13^

Given the macroscopic instability of both suspensions observed on day 60 and the inconsistent particle size results on days 45 and 60, it can be inferred that these aspects may be interrelated. The challenges in resuspending the samples may impact the accuracy of particle size measurements. Over time, suspensions can form compact sediments that are difficult to redisperse manually. This phenomenon is primarily due to the natural effect of gravity acting on the particles in suspension.^39^ The formation of such irredeemable sediments is referred to as “caking”. Consequently, the observed decrease in particle size over time may reflect the measurement of smaller particles that remain suspended or are partially redispersible, as numerous particles settle at the bottom of the storage vial.

This instability could potentially be mitigated by simulating the patient’s daily routine, such as opening the flasks and withdrawing 1 mL of the suspension each day. Alternatively, optimization of the vehicle could help, for example, by incorporating zeta potential modulators or adjusting the vehicle’s viscosity.^40,41^ Additionally, the high standard deviation in particle size over time may be due to inconsistent shaking, since the process is performed manually and may vary even when done by the same analyst. This variability reflects the procedures commonly carried out by patients at home, as well as by nurses and pharmacists in hospital settings.^18,41^

#### Drug Content

Given the critical need for alternative dosage forms for newborns diagnosed with CT, healthcare professionals are increasingly tasked with adapting commercially available tablets into liquid preparations suitable for infants. This adaptation process presents several challenges, including the risk of microbial contamination, excipient–excipient and drug-excipient incompatibilities, as well as physical and chemical instability, which may lead to inaccurate dosing. These factors underscore the importance of this study, particularly regarding the stability of pyrimethamine in extemporaneous oral suspensions.

The chemical stability of a drug is typically assessed based on its degradation rates over time, using the initial drug content postformulation as a reference point. In this study, acceptable drug content limits were established according to the USP which stipulates that drug content should be maintained within 90–110% of the initial concentration.^18^

Although dose uniformity was not directly assessed using compendial methods (e.g., content uniformity or dosage unit uniformity), this approach is consistent with extemporaneous suspensions, for which no specific pharmacopeial methodology or suitable comparator product is available. In such systems, dose reproducibility is intrinsically dependent on adequate redispersion prior to administration.^8,9^ Therefore, drug content was monitored over time as an indirect indicator of dose consistency the drug content analysis over time for Suspensions X and S are illustrated in Figure 5.

**Figure 5:**
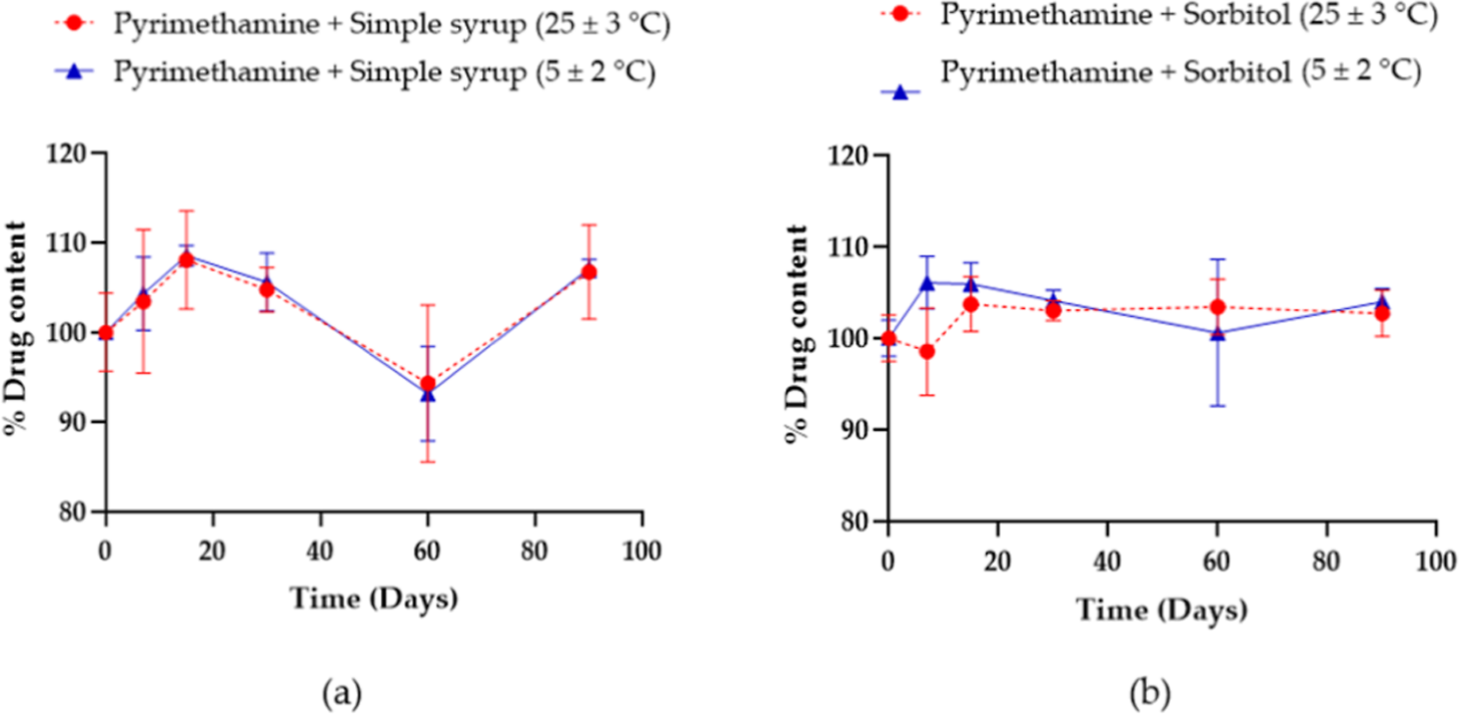
Drug content variation of extemporaneous pyrimethamine formulations over 90 days, stored under two temperature conditions: (a) simple syrup as the vehicle and (b) sorbitol solution as the vehicle. Samples were maintained at 25 ± 3 °C (dashed lines) and 5 ± 2 °C (solid lines). Data are presented as mean ± standard deviation (*n* = 3).

Pyrimethamine content remained within the 90–110% acceptance range throughout the study, in accordance with USP guidelines.^18^ Despite the overall stability, significant deviations in drug content percentages were noted for both suspensions. These deviations can be attributed to the sampling process, where homogeneous sampling is crucial for accurately measuring pyrimethamine content. Irregular hand shaking and variations in the amount of precipitate during sampling may have compromised precision. This is supported by the observation that final concentrations were similar to initial concentrations, despite the outliers recorded throughout the experiment. Such limitations are particularly relevant in extemporaneous suspensions, where the uneven distribution of excipients from the tablets complicates visual inspection during the manual homogenization process.^42^

The chemical stability of a drug over time is a critical factor for product approval, as drug content directly influences treatment efficacy. In the case of extemporaneous formulations, a lack of established stability parameters could result in inaccurate dosage regimens, thereby undermining treatment effectiveness.^10,43^ Consequently, the technical and scientific data regarding drug stability from these formulations are essential for developing effective action plans for preparing extemporaneous suspensions, ensuring safer and more effective therapy.

The findings are particularly promising when compared to currently used alternatives for extemporaneous suspensions. Hospitals frequently rely on commercially available suspending vehicles, such as Ora-Plus and Ora-Sweet. Unlike the proposed vehicles of 70% sorbitol and 85% sucrose, which are low-cost options, Ora-Plus and Ora-Sweet are industrial products priced significantly higher approximately USD 60 to USD 80 per 500 mL making them around 20 times more expensive than the vehicles explored in this work.^14,19^ This high cost poses a significant barrier for developing countries with a high incidence of CT cases.

Additionally, previous studies have demonstrated that pyrimethamine suspensions formulated with Ora-Plus and Ora-Sweet exhibit stability similar to that observed in this research, remaining chemically stable for 48 and 90 days, respectively, stored in amber bottles at room temperature and refrigeration.

### Microbiological Analyses

Microbiological analyses were conducted according to acceptance criteria allowing a limit of up to 100 CFU/mL, with expected values of up to 16 CFU/mL for TSA and SA, and 64 CFU/mL for TAT broth. From T0 to T30, no microbial growth was detected in the formulations, meeting the initial treatment period recommended for congenital toxoplasmosis. At 45 days (T45), microbial growth exceeding the permissible limit was observed only in the formulation with 85% simple syrup, possibly due to localized contamination during handling, without compromising the overall safety of the formulation.^44^

At 60 (T60) and 90 (T90) days, no significant microbial growth (>1 CFU/mL) was observed, indicating prolonged microbiological stability. The presence of *Escherichia coli* was assessed at all time points, but no characteristic colonies were detected, confirming the absence of the pathogen in the formulations within the detection limits of 32 CFU/mL and 8 CFU/mL, respectively. These results demonstrate that extemporaneous pyrimethamine suspensions, when prepared under controlled conditions, maintain adequate microbiological stability, meeting safety criteria required for the treatment of pediatric patients with congenital toxoplasmosis.

The comparative analysis between the suspensions developed in this study and those formulated with industrially produced vehicles demonstrates that extemporaneous pyrimethamine suspensions can be effectively prepared using low-cost excipients, maintaining satisfactory stability for up to 45 days for Suspension S and 60 days for Suspension X, at both refrigeration and room temperature conditions. This finding reinforces the feasibility of accessible pharmaceutical alternatives that support improved therapeutic adherence, efficacy, and safety in the treatment of congenital toxoplasmosis in children, particularly in resource-limited settings.

## Conclusions

In conclusion, a robust HPLC method was adapted and validated for the quantification of pyrimethamine in both formulations. The study demonstrates that extemporaneous pyrimethamine suspensions, prepared with sucrose and sorbitol as vehicles, remain stable for up to 45 days for Suspension S and 60 days for Suspension X, at both temperature conditions. Assay, pH values, particle size, and microbiological quality remained within acceptable limits for oral administration, with pyrimethamine concentrations consistently maintained throughout the study. Although strong manual shaking is necessary to ensure proper redispersion due to particle aggregation, the formulations showed no significant microbial growth over time, supporting their microbiological stability. These findings provide guidance for healthcare professionals preparing cost-effective extemporaneous pyrimethamine formulations for the treatment of congenital toxoplasmosis in pediatric patients.

## Materials and Methods

### Materials

Pyrimethamine tablets 25 mg were produced by Farmoquímica S/A (Rio de Janeiro, Brazil) and contained ethanol, magnesium stearate, starch, sodium ducosate, lactose monohydrate, and water as excipients. Pyrimethamine reference standard was acquired from Sigma-Aldrich (São Paulo, Brazil). Methanol (HPLC) was purchased from JT Baker. O-Phosphoric acid and potassium phosphate monobasic anhydrous were obtained from Pro-análise (Porto Alegre, Brazil) and Synth (São Paulo, Brazil), respectively. Ultrapure water was produced using Milli-Q Integral 5 (Bay City, USA). Sucrose was acquired from Camil Alimentos (São Paulo, Brazil) and sorbitol from SM Empreendimentos Farmaceuticos Ltd. (São Paulo, Brazil).

## Preparation of Suspensions

Pyrimethamine content in the commercial tablets was quantified via spectrophotometry, in accordance with USP guidelines. The tablets yielded a pyrimethamine content of 99.71%, falling within the pharmacopeial requirements of 93–107%.^45^ Therefore, the suspensions were prepared at a pyrimethamine concentration of 2 mg/mL by triturating 25 mg pyrimethamine tablets and dispersing the powder in one of two vehicles 70% sorbitol (w/v; Suspension S) or 85% sucrose (w/v; Suspension X) in accordance with the method of Soares Rodrigues Costa et al. (2019).^26^ Batches of 600 mL final volume were prepared, obtained by crushing 48 pyrimethamine tablets. Briefly, tablets were weighed and ground to a fine powder using a mortar and pestle. One-third of the vehicle was then added to the powder and mixed to form a uniform paste; the remaining vehicle was gradually incorporated, and the blend was homogenized until the suspensions appeared visually uniform. Finished preparations were transferred to amber bottles and stored at 25 ± 2 °C and 5 ± 3 °C for subsequent stability testing.

### Chromatographic Conditions

The chromatographic conditions for pyrimethamine quantification were established using high-performance liquid chromatography (HPLC) (Chromaster VWR Hitachi). The system was equipped with a degasser, quaternary pump 5160, autosampler 5260, column oven 5310, and DAD 5430 detector. A Hypersil Gold analytical column (250 mm × 4.6 mm × 5.0 μm, Thermo, USA) was used. The isocratic method used a mobile phase consisting of potassium phosphate buffer pH 2,6 and methanol (60:40, v/v), degassed, and vacuum filtered through a 0.22 μm membrane prior to use, with a flow rate of 1.0 mL/min. The injection volume was 20 μL, and the run time was set to 15 min. The column oven was maintained at 25 °C, and detection was performed at a wavelength of 235 nm. The EzChrom Elite software, version 3.3.2 SP2 (Hitachi), was used to process the data.

## Method Validation

The analytical method was validated according to the International Conference on Harmonization (ICH Guideline Q2­(R2)) guidelines^25^ for specificity, linearity, accuracy, precision (including repeatability and intermediate precision), and ICH Guideline Q14 for robustness^46^. A test sample containing pyrimethamine at a final concentration of 16 μg/mL was prepared and used throughout the validation process and stability study. Prior to sampling, the bottles containing the pyrimethamine suspensions were vigorously homogenized using circular manual agitation for 5 min to ensure uniform dispersion. An aliquot of the suspension, in the middle of the bottles, equivalent to 4 mg of pyrimethamine was transferred into a 25 mL volumetric flask. Methanol was added, and the mixture was sonicated for 5 min. After cooling to room temperature, the solution was diluted to volume with methanol. Immediately prior to injection into HPLC, the samples were filtered through a 0.45 μm PTFE syringe filter directly to a vial.

Specificity was assessed under the given HPLC conditions using the following samples: pyrimethamine standard solution, test samples (suspension containing pyrimethamine), suspension vehicles, and diluent. Pyrimethamine solutions were subjected to forced degradation (1 and 0.1 M base and acid hydrolysis, oxidation, photolysis, and thermal degradation at 50 °C) to evaluate the method’s specificity against potential degradation products. Peak purity was determined using a photodiode array detector, and the absence of coelution was confirmed with an acceptance criterion of purity peak higher than 0.9990.

Linearity was evaluated by constructing three analytical curves using pyrimethamine reference standard concentrations ranging from 80% (12.8 μg/mL) to 120% (19.2 μg/mL) in methanol. Linearity was confirmed through linear regression analysis previously by a homoscedasticity evaluation. The coefficient of variation was analyzed to verify the randomness of errors and was expected a value greater than 0.99. Further, residuals were examined to ensure that no trend formation occurred in the analytical data.

Precision was assessed as both repeatability and intermediate precision, using a placebo solution spiked with pyrimethamine at (0.0128 mg/mL, 0.0144 mg/mL, 0.0160 mg/mL, 0.0176 mg/mL, 0.0192 mg/mL) concentrations within the linearity range. Repeatability was evaluated by the same analyst, while intermediate precision was assessed by two different analysts on different days. A minimum relative standard deviation (RSD) of no more than 2.0% was considered acceptable for both repeatability and intermediate precision.

Accuracy was tested in triplicate at 80% (12.8 μg/mL), 100% (16.0 μg/mL), and 120% (19.2 μg/mL) of the target concentration, totaling nine measurements. Recovery was calculated by comparing the measured concentrations with theoretical values, and an accuracy deviation of no more than 2.0% was considered acceptable.

Robustness was tested on both standard and sample solutions by varying conditions such as mobile phase pH (2.5 and 2.7), flow rate (0.9 and 1.1 mL/min), and mobile phase composition (58:42 and 62:38). The criteria for acceptance were: theoretical plates greater than 4000, tailing factor less than 2, capacity factor and resolution greater than 2 and RSD no more than 2%.

The evaluation of potential drug adsorption onto the filter material was performed during method suitability assessment. Given the complex nature of the sample matrix (suspension), filtration was considered mandatory. Volumes of up to 6 mL were evaluated for both standard and sample solutions. The results demonstrated that, for both matrices, the type of filter employed did not affect drug quantification, with adsorption values lower than 0.1%.

### Characterization and Stability Study

The characterization and stability study of each suspension was made by storing the samples in triplicate at 25 ± 2 °C and 5 ± 3 °C for 90 days. Pyrimethamine drug content was determined throughout periodically (on days 0, 2, 7, 15, 30, 45, 60, and 90) for stability study using a triplicate experimental design at each time point. For each sampling interval, three independently prepared sample solutions of 16 μg/mL, as described in Method Validation, were analyzed. Each replicate solution was injected once into the HPLC system, and the results were expressed as the mean of the three determinations.

The stability study evaluated the following physicochemical parameters over time: color/appearance, odor, pH, particle size, and drug content. pH measurements were conducted at 25 °C using a digital pH meter (TEC-3MP, Tecnal, Brazil).

Particle size was determined using a Particle Size Analyzer (PSA1190, Anton Paar, Austria) in Fraunhofer mode, with measurements taken in a liquid medium (water) under ultrasound at a rate of 250 rpm, for a duration of 1 min, with a pump rate of 120 rpm and obscuration of 10%. Water was used as the dispersant. The instrument required an obscuration level between 5 and 30%; therefore, the amount of sample added did not interfere with the effective dilution once the target obscuration range was achieved. For standardization purposes, measurements were initiated when an obscuration value of 10% was reached.

Drug content was monitored over time using the previously described HPLC method. According to the United States Pharmacopoeia,^18^ acceptable drug content results were defined as a percent recovery between 90% and 110% relative to the initial value at time zero (T 0 h).

#### Microbiological Stability Study

For microbiological evaluation, sterilized culture media were used, including Tryptone-Azolectin-Tween (TAT) broth, Trypticase Soy Agar (TSA), Sabouraud agar (SA), Tween 20 (as neutralizer), MacConkey broth, and MacConkey agar. Analyses were conducted on days 0, 2, 7, 15, 30, 45, 60, and 90, with storage at room temperature (22.5 °C). Microbial counts followed the acceptable limits for nonsterile products: ≤10^2^ CFU/mL for bacteria, ≤10^1^ CFU/mL for fungi, and absence of *E. coli* in 1 mL.^47^

Total microbial count was performed from 1:10 dilutions, with each 1 mL plated onto TSA and SA using the pour-plate technique, followed by incubation for 5–7 days at temperatures ranging from 20 to 25 °C to 30 to 35 °C. For *E. coli* detection, 1 mL of a 1:100 dilution was incubated in MacConkey broth, followed by subculturing onto MacConkey agar and incubation at 43 °C for 24 h. The presence of red colonies was indicative of contamination.

### Statistical Analysis

Statistical analyses were performed using two-way ANOVA followed by Bonferroni’s post hoc test. Results are expressed as the mean ± standard deviation of triplicate determinations. Differences were considered statistically significant at *p* < 0.05. All analyses were conducted using GraphPad Prism 6 software (GraphPad Software, Inc., San Diego, CA, USA).
